# Factors Associated with Maternal Worry about Her Young Child Exhibiting Choosy Feeding Behaviour

**DOI:** 10.3390/ijerph15061236

**Published:** 2018-06-12

**Authors:** Pauline M. Emmett, Nicholas P. Hays, Caroline M. Taylor

**Affiliations:** 1 Centre for Child and Adolescent Health, Bristol Medical School, University of Bristol, Bristol BS8 2BN, UK; caroline.m.taylor@bristol.ac.uk; 2 Nestlé Product Technology Center—Nutrition, 1814 La Tour-de-Peilz, Switzerland; nicholaspaul.hays@nestle.com

**Keywords:** choosiness, complementary feeding, feeding behaviour, ALSPAC, pre-school children, parental feeding practices

## Abstract

Choosiness in young children is a normal behaviour that sometimes worries parents. The study aimed to investigate factors that are associated with a mother being worried about her child’s choosy feeding behaviour. Parents of singleton children from the Avon Longitudinal Study of Parents and Children (*n* = 5710) completed a questionnaire assessing perception of their child’s choosy feeding behaviour at 15 months of age and whether this choosiness worried them. Feeding behaviours and practices throughout the first 15 months were captured. Multinomial logistic regression models with three levels of worry (*not at all*, *a bit* and *greatly*) as the dependent variables tested associations with variables from pregnancy and infancy. Half of the children (56%) were described as choosy at 15 months; of these 27% had mothers who were *a bit* worried and 5% *greatly* worried. Mothers showed greater odds of being worried if the child was first born, difficult to feed or refused solids by 6 months of age. Worried mothers had shown greater odds of introducing lumpy foods late (after 9 months). Feeding vegetables regularly by 6 months was associated with lower odds of worry at 15 months. Support and advice to parents at the start of complementary feeding could help to alleviate worry. Parents should be reassured that choosiness is a normal part of child development.

## 1. Introduction

During the introduction of complementary foods infants learn to consume new tastes and textures and some children find this more difficult than others. Parents play a crucial role in providing a supportive environment during this phase of a child’s life [1]. However, parents do not always find this an easy process and by the age of 3 years many children are perceived as ‘picky eaters’ by their parents [2]. The parent being worried by their child being choosy with regard to foods in toddlerhood has been shown to be highly associated with the child being perceived as a picky eater at age 3 years [3] and this has been associated with a diet low in dietary fibre [4], fruit, vegetables and meat [5]. In other studies, parental worry has been investigated only in relation to the perception that the child is under-eating [6] or underweight [7], not in relation to child choosiness or picky eating. It is important to understand how the parent-child relationship around feeding develops during the early years because this is likely to determine whether the child eats a balanced diet during childhood and beyond [1].

The aim of this study is to investigate factors associated with maternal worry about her child’s choosiness at 15 months of age. The factors to be explored include maternal anxiety and depressive symptoms, parity, feeding behaviours during infancy and complementary feeding practices.

## 2. Materials and Methods

The Avon Longitudinal Study of Parents and Children (ALSPAC) is a population-based prospective study researching environmental and genetic influences on childhood health, behaviour and development longitudinally. All pregnant women living in a prescribed area of the former county of Avon in south-west England with an expected delivery date between April 1991 and December 1992 were eligible; 14,541 pregnant women were enrolled, resulting in 14,062 live births with 13,988 alive at 1 year of age [8,9]. Further information about ALSPAC is available at www.bris.ac.uk/alspac and the study website contains details of all the data that are available through a fully searchable data dictionary (http://www.bris.ac.uk/alspac/researchers/data-access/data-dictionary). Ethics approval for the study was obtained from the ALSPAC Ethics and Law Committee and the Local Research Ethics Committees. The primary data collection was through self-completion postal questionnaires.

### 2.1. Maternal Worry about Child Choosiness

The primary caregiver (usually the mother) received a questionnaire about her child aged 15 months. One question asked: ‘Since your child was 6 months old has he/she at any time been choosy with food?’ and if the answer was yes, she was asked if this behaviour worried her greatly, a bit or not at all.

### 2.2. Complementary Feeding and Child Feeding Behaviour

Three questionnaires including questions on complementary feeding and child feeding behaviour in infancy were completed by the mother when the child was 4 weeks, 6 months and 15 months old. The full questionnaires are available from the study website (http://www.bristol.ac.uk/alspac/researchers/resources-available/data-details/questionnaires/). The wording of the questions and frequency of the answers used in this analysis are shown in Table 1.

### 2.3. Maternal, Pregnancy and Background Variables

Maternal and demographic variables were obtained from postal questionnaires in pregnancy and after the birth of the child. These included parity (0, 1, ≥2), maternal age at delivery (≤20, 21–25, 26–30, >30 years of age) and highest educational attainment grouped into five categories (None; Vocational; Ordinary Level Certificate of School Education usually taken at 16 years of age; Advanced Level Certificate usually taken at 18 years of age; Degree). Pre-pregnancy body mass index, from self-reported pre-pregnancy weight and height, was grouped as <20, 20–24.99, 25–29.99 and ≥30 kg/m^2^.

Measures of maternal anxiety were obtained using the Crown–Crisp anxiety subscale (scale 0–16) [10] and of maternal depression using the Edinburgh Postnatal Depression Scale (scale 0–29) [11,12]. Both scores were collected at 18 and 32 weeks of pregnancy, and at 2 and 8 months postpartum. High levels of symptoms were a score of ≥9 for anxiety and ≥13 for depression. 

The sex and birth weight of the child (grouped as ≤2500, 2501–3000, 3001–3500, 3501–4000, >4000 g) were obtained from medical records.

### 2.4. Statistical Analysis

Statistical analysis was carried out with SPSS v23.0 (IBM Corp, Armonk, NY, USA) on mothers of singleton children who were described as choosy.

A multinomial logistic regression model with the degree of maternal worry as the dependent variable included demographic and perinatal variables (age and educational status of the mother, parity, sex of the child and birth weight (grouped)) as predictors. Maternal anxiety and depressive symptoms at each age measured were included along with factors occurring in the first year of life: weak sucking, choking, slow feeding, taking small quantities and difficulty to feed at 4 weeks, duration of breastfeeding, age of introduction to solid foods, choking, refusal of solid foods, frequency of consumption of commercial baby foods and family foods including raw fruit and fresh vegetables, difficulty feeding at 6 months and age at introduction of lumpy foods between 6 and 15 months.

## 3. Results

Of the children studied at 15 months (*n* = 5701) 56% had mothers who described them as being choosy with food (*n* = 3214). These mothers were asked if the choosiness worried them 27% indicated that they were *a bit* worried and 5% were *greatly* worried. Factors potentially associated with worry were investigated in these 3214 mothers of choosy children using a regression model. The model explained 13.8% of the variance in the degree of worry the mother had about her child’s choosiness at 15 months of age (Table 2). The strongest associate with worry was the child being first-born, with the mother having more than twice the odds of being *greatly* worried about choosiness as for children who were not first-born. There was no association with sex, birthweight of the child or age of the mother at the birth. Being *greatly* worried was not associated with maternal education but being *a bit* worried was: the highest educated mothers showed the greatest odds of being *a bit* worried (odds ratio (OR) 1.61, 95% confidence interval (CI) 1.14, 2.29; *p* = 0.007).

Of the factors assessed when the child was 4 weeks of age, weak sucking, choking and slow feeding were not associated with the mother being *greatly* worried, but the child taking small quantities of feed was associated with *greater* worry. The mother finding the child difficult to feed was associated only with her being *a bit* worried.

The child’s feeding behaviour up to and including 6 months of age showed some significant associations. The mother finding the child difficult to feed and the child refusing solids were both associated with increased odds of the mother being either *a bit* or *greatly* worried about choosiness. However, there was no association with the age of introduction of solids or the child being fed on demand, being a slow feeder, taking small quantities of feed or choking.

In comparison with being breast fed for ≥6 months there were no associations with never being breast fed; there was, however, a suggestion that some breast feeding for <3 months was associated with decreased odds of the mother being *a bit* worried (*p* = 0.048). When investigating the foods fed to the child at 6 months if the child was fed fresh vegetables regularly the child’s mother showed lower odds of being worried at the child’s choosiness. The final significant findings were the increased odds of late introduction of ‘lumpy’ foods to children whose mothers worried about choosiness.

There were no associations of worry about choosiness with symptoms of depression or anxiety in the mothers either in pregnancy or the first year postpartum.

## 4. Discussion

Choosiness with food is a normal part of child development with a high prevalence in the second year of life where in evolutionary terms it may have conveyed an advantage in limiting the likelihood of a child eating harmful substances. However, parents are often worried by this behaviour in their children and we have previously shown that 56% of children were identified as being choosy at 15 months and of these if the mother was greatly worried half went on to be described as *very* picky eaters at 3 years, compared with only 17% if the mother was not worried by the choosiness [3]. The current study found that the child being first-born is associated with maternal worry and that difficulty feeding the child even as early as 4 weeks of age can increase the odds of later worry. The child refusing solids and the parents having difficulty with feeding at 6 months are associated with later worry about choosiness. Feeding practices such as not feeding vegetables at 6 months of age and delaying the introduction of lumpy foods to beyond 9 months of age are also associated with worry about choosiness. These findings suggest that children could benefit if parents, particularly first-timers, were given support around the time of the introduction of complementary foods.

Few studies have investigated difficulty with feeding and parental perception of “picky eating” in representative samples of children under 2 years of age. However, the Feeding Infants and Toddlers Study (FITS) in the USA collected cross-sectional information from more than 3000 parents, sampled across the whole country, about whether they considered their child to be a “picky eater” at ages between 4 and 19 months [13]. The prevalence of picky eating rose from 19% in 4–6-month-olds to 46% in 15–18-month-olds and 50% in 19–23-month-olds. In a Chinese study of 1414 children from 8 different cities the prevalence of picky eating was 12% in 6–11-month-olds and 36% in 24–35-month-olds [14]. In our study parents were not asked about their child’s picky eating behaviour; however, comparison can be made with FITS in that in our study 22% had refused solids by 6 months and 56% were considered “choosy” by parents at 15 months, these being somewhat similar proportions. Both FITS and the Chinese study collected a single 24-h recall of diet in the children and found no differences in foods eaten between the picky and non-picky children [13,14] unlike in our study where we assessed frequency of consumption of certain foods by questionnaire. However, our findings align with those of the Generation R study in the Netherlands which found that children introduced to vegetables before 6 months had a lower food fussiness score at 4 years of age than those introduced after 6 months [15]. The FITS study investigated picky eating behaviour in more depth in older children and found that children who were considered *somewhat* picky or *very* picky by parents were much more likely to resist new foods or refuse certain food textures than non-picky children, our study showed similar traits to these [16].

Parental worry about feeding their child has not been widely studied especially in toddlerhood. A cross-sectional study of 4–8-year-olds by Brown et al. in the USA [6] has shown that maternal worry about a child under-eating was associated with more *pressure to eat* and the greater use of *bribery* to persuade the child to eat. Greater food fussiness but not neophobia in the child was associated with increased odds of the mother being worried about the child under-eating. Similar findings were made by Gregory et al. [7] in 2–4-year-olds in Australia. Child food fussiness was associated with the mother using *pressure to eat*; however, mothers’ worry about the child being underweight was a much stronger associate of the application of *pressure to eat*. In our study we did not have a measure of *pressure to eat* but it seems likely that worried parents may have used this tactic when feeding their choosy child. Certainly, in the Generation R study, where nearly 5000 mother–child pairs were followed, child fussiness at 3 years was associated with higher levels of parental *pressure to eat* one year later and increased levels of fussy eating 2 years after that [17].

We have found previously that late introduction to lumpy foods was associated with an increase in parentally perceived feeding difficulties from 39% at 6 months to 52% at 15 months [18]. The children introduced late to lumps ate fruit and vegetables less often and showed twice the odds of having definite likes and dislikes compared with children for whom lumpy foods were introduced at the recommended age. In a follow-up of these children at age 7 years those introduced late to lumps continued to have lower odds of eating all types of fruits and vegetables and greater odds of being choosy with food [19].

We found no associations of mothers’ worry with mothers’ symptoms of depression or anxiety. To our knowledge, this has not been researched before and it seems likely from our results that a mother’s worry about her child’s choosiness was not generally associated with symptoms of these mental health problems. 

The strengths of this study include: (i) using a question about child choosiness that also asked about whether this worried the mother; (ii) parental questionnaires were completed prospectively during the complementary feeding process and therefore not subject to recall bias; (iii) a large number of participants were followed. Limitations include: (i) some attrition and incomplete data collection; (ii) data were collected from untrained participants by postal questionnaires and therefore may be biased by lack of understanding or varying interpretation of and response to the questions; (iii) the study was carried out in one area of the UK in 1990s, which may limit generalizability; (iv) complementary feeding advice has changed from introduction of solids around 4 months of age to around 6 months of age, since this study was completed. Further research is needed to assess the effect of this change and should include questions about parental worry.

## 5. Conclusions

This study provides evidence that child feeding behaviours in infancy can lead to worry in parents that may affect how they feed their child. First time parents are more prone to worry. Support and advice to parents at the time of starting complementary feeding could help to allay fears and allow them to be more relaxed about the natural choosy phase that many children go through in toddlerhood.

## Figures and Tables

**Table 1:** Questions asked to parents, about feeding their child at different ages, together with the answer categories used in the regression models, grouped as shown for analysis, with frequency of occurrence in the whole cohort.

Questions as Asked	Answer Categories	Overall Percent
**Child aged 4 weeks**		
Please indicate if your baby has had the following feeding behaviours:		
(a) Weak sucking	Always/Sometimes	8.9
	Occasionally/No not at all	91.1 (Reference)
(b) Choking	Always/Sometimes	26.5
	Occasionally/No not at all	73.5 (Reference)
(c) Slow feeding	Always/Sometimes	24.7
	Occasionally/No not at all	75.3 (Reference)
(d) Taking only small quantities at each fed	Always/Sometimes	29.1
	Occasionally/No not at all	70.9 (Reference)
Do you feel your baby is difficult to feed?	Yes, very/Yes, quite difficult	11.4
	No, not difficult	88.6 (Reference)
**Child aged 6 months**		
Is the baby fed ‘on demand’, i.e., whenever he/she is hungry?	Always/Sometimes	83.2
	No not at all	16.8 (Reference)
Please indicate if your baby had any of the following feeding behaviours and when they occurred:		
(a) Slow feeding	Yes 0–3 months/Yes 4–6 months	28.9
	No not at all	71.1 (Reference)
(b) Choking	Yes 0–3 months/Yes 4–6 months	22.3
	No not at all	77.7 (Reference)
(c) Taking only small quantities at each fed	Yes 0–3 months/Yes 4–6 months	33.8
	No not at all	66.2 (Reference)
Do you feel you have ever had any difficulty feeding your baby?	Yes, great/Yes, some difficulties	35.4
	No, no difficulties	64.6 (Reference)
Has your baby refused to take solids before 6 months of age	Yes	21.6
	No	78.4 (Reference)
Breast feeding duration	Never	21.4
	<3 months	22.6
	3–5 months	17.3
	6 months or more	38.7 (Reference)
Age solid foods introduced	0–3 months	71.9
	4 months	25.0
	5 months or more	3.1 (Reference)
Ready-prepared baby foods (from a jar, tin or packet) at 6 months	Not answered	6.7
	22 times or more per week	11.5
	15–21 times per week	21.6
	8–14 times per week	32.1
	1–7 times per week	20.0
	None	8.1 (Reference)
Vegetables eaten at 6 months (home-prepared)	Not answered	4.7
	8 times or more per week	12.2
	7 times per week	19.8
	1–6 times per week	44.9
	None	18.4 (Reference)
Fresh fruit eaten at 6 months	Not answered	1.6
	7 times or more per week	6.4
	1–6 times per week	36.0
	None	56.0 (Reference)
Babies first solid meals are usually a puree. When did your child first start having meals with lumps in?	Before 6 months	11.9
	Between 6 and 9 months	70.2 (Reference)
	10 months or more	17.9

**Table 2:** Factors associated with maternal worry about her child being choosy at 15 months of age.

Not Worried (*n* = 2182) Variable (Reference Category)		Mother *A Bit* Worried at 15 Months (*n* = 872)			Mother *Greatly* Worried at 15 Months (*n* = 160)		
		OR	95% CI	*p* Value	OR	95% CI	*p* Value
Parity (third-born or more)	1st born	**1.82**	**1.40, 2.36**	**<0.001**	**2.14**	**1.25, 3.68**	**0.006**
	2nd born	1.13	0.87, 1.47	0.35	0.75	0.42, 1.35	0.34
Child age 4 weeks							
Weak sucking (no)	Yes	0.87	0.65, 1.17	0.37	0.63	0.36, 1.11	0.11
Choking (no)	Yes	1.05	0.87, 1.27	0.59	1.43	0.99, 2.07	0.060
Slow feeding (no)	Yes	0.97	0.78, 1.20	0.75	1.26	0.83, 1.91	0.27
Small quantities (no)	Yes	1.15	0.95, 1.39	0.17	**1.54**	**1.05, 2.26**	**0.027**
Difficult to feed (no)	Yes	**1.40**	**1.08, 1.82**	**0.012**	1.12	0.67, 1.85	0.67
Age solids introduced (≥5 months)	0–3 months	1.11	0.68, 1.79	0.68	1.43	0.42, 4.83	0.56
	4 months	1.09	0.67, 1.78	0.34	1.22	0.35, 4.21	0.75
Child age 6 months							
Fed on demand (no)	Yes	0.96	0.76, 1.22	0.73	0.84	0.52, 1.34	0.45
Difficulty to feed (no)	Yes	**1.47**	**1.23, 1.76**	**<0.001**	**1.53**	**1.05, 2.24**	**0.027**
Slow feeding (no)	Yes	0.99	0.81, 1.22	0.96	1.32	0.87, 1.99	0.16
Small quantities (no)	Yes	1.07	0.89, 1.30	0.47	1.11	0.75, 1.65	0.59
Choking (no)	Yes	1.20	0.99, 1.46	0.06	1.41	0.96, 2.06	0.08
Refused solids (no)	Yes	**1.46**	**1.21, 1.77**	**<0.001**	**1.92**	**1.33, 2.76**	**0.001**
Baby food (none)	Not answered	1.05	0.65, 1.69	0.86	1.10	0.36, 3.32	0.87
	22 times +/week	1.30	0.86, 1.97	0.21	2.01	0.84, 4.84	0.12
	15–21 times/week	**1.50**	**1.03, 2.18**	**0.035**	1.35	0.57, 3.19	0.50
	8–14 times/week	1.15	0.80, 1.65	0.46	1.38	0.60, 3.15	0.45
	1–7 times/week	1.11	0.76, 1.62	0.58	1.09	0.46, 2.61	0.84
Vegetables eaten (none)	Not answered	**0.62**	**0.38, 1.00**	**0.048**	**0.28**	**0.08, 0.97**	**0.044**
	8 times +/week	0.80	0.58, 1.11	0.12	**0.42**	**0.20, 0.89**	**0.023**
	7 times/week	**0.73**	**0.55, 0.97**	**0.032**	**0.51**	**0.28, 0.93**	**0.029**
	1–6 times/week	0.87	0.69, 1.08	0.20	0.76	0.50, 1.15	0.19
Fruit eaten (none)	Not answered	1.40	0.72, 2.71	0.32	0.61	0.08, 4.85	0.64
	7 times +/week	0.73	0.50, 1.08	0.11	1.29	0.61, 2.72	0.50
	1–6 times/week	0.94	0.79, 1.13	0.52	0.84	0.57, 1.24	0.39
Duration of breast feeding (≥6 months)	Never	0.80	0.62, 1.03	0.09	0.80	0.48, 1.35	0.41
	<3 months	**0.79**	**0.63, 1.00**	**0.048**	0.89	0.55, 1.42	0.62
	3–5 months	0.81	0.63, 1.03	0.08	0.92	0.55, 1.52	0.74
Age introduced to lumps (6–9 months)	<6 months	0.89	0.66, 1.19	0.42	0.74	0.56, 1.53	0.42
	>10 months	**1.43**	**1.16, 1.76**	**0.001**	**2.33**	**1.60, 3.39**	**<0.001**
Maternal anxiety symptoms							
18 weeks gestation (no)	Yes	1.10	0.81, 1.47	0.55	1.39	0.80, 2.39	0.24
32 weeks gestation (no)	Yes	1.32	0.99, 1.76	0.064	1.03	0.58, 1.82	0.93
2 months postpartum (no)	Yes	1.25	0.87, 1.79	0.23	1.43	0.76, 2.69	0.26
8 months postpartum (no)	Yes	1.24	0.87, 1.76	0.23	1.56	0.85, 2.89	0.16
Maternal depressive symptoms							
18 weeks gestation (no)	Yes	0.92	0.66, 1.28	0.62	0.75	0.40, 1.40	0.37
32 weeks gestation (no)	Yes	1.05	0.76, 1.44	0.78	1.05	0.58, 1.91	0.87
2 months postpartum (no)	Yes	0.91	0.63, 1.31	0.60	1.28	0.69, 2.39	0.44
8 months postpartum (no)	Yes	0.92	0.63, 1.34	0.65	1.50	0. 80, 2.84	0.21

Reference: Mother not worried about child being choosy at 15 months. Model explains 13.8% of the variance (*n* = 3214).
